# Doxycycline Malaria Prophylaxis Impact on Risk of Travelers’ Diarrhea among International Travelers

**DOI:** 10.4269/ajtmh.20-0241

**Published:** 2020-08-17

**Authors:** Kathryn Lago, Kalyani Telu, David Tribble, Anuradha Ganesan, Anjali Kunz, Charla Geist, Jamie Fraser, Indrani Mitra, Tahaniyat Lalani, Heather C. Yun, for the Infectious Disease Clinical Research Program TravMil Study Group

**Affiliations:** 1 Brooke Army Medical Center, Fort Sam Houston, Texas;; 2 Uniformed Services University of the Health Sciences, Bethesda, Maryland;; 3 Infectious Disease Clinical Research Program, Department of Preventive Medicine and Biostatistics, Uniformed Services University of the Health Sciences, Bethesda, Maryland;; 4 The Henry M. Jackson Foundation for the Advancement of Military Medicine, Inc., Bethesda, Maryland;; 5 Walter Reed National Military Medical Center, Bethesda, Maryland;; 6 Madigan Army Medical Center, Tacoma, Washington;; 7 Landstuhl Regional Medical Center, Landstuhl, Germany;; 8 Naval Medical Center, Portsmouth, Virginia

## Abstract

International travelers are frequently at risk for travelers’ diarrhea (TD) and malaria. Doxycycline was one of the earliest antibiotics shown to have efficacy in TD prevention. With increasing resistance and recommendations against antibiotic chemoprophylaxis, doxycycline fell out of use. We evaluated TD incidence and risk factors in a prospective cohort of travelers, specifically in regard to malaria prophylaxis. Travelers’ diarrhea was defined as ≥ 3 loose stools in 24 hours or two loose stools in 24 hours associated with other gastrointestinal symptoms. The Poisson regression model with robust error variance was used to estimate the RR of TD. Three thousand two hundred twenty-seven trips were enrolled: 62.1% of participants were male, with a median age of 39 years (interquartile range [IQR] 27,59) and a median travel duration of 19 days (IQR 12,49); 17.4% developed TD; 32% traveled to Africa, 40% to Asia, and 27% to the Caribbean and Latin America; and 20% took doxycycline for malaria chemoprophylaxis, 50% took other antimalarials, and 30% took none. Decreased RR of TD was associated with doxycycline (RR 0.62 [0.47–0.82], *P* < 0.01) and military travel (RR 0.57 [0.47–0.70], *P* < 0.01). Increased risk of TD was associated with female gender (RR 1.28 [1.09–1.50], *P* < 0.01), hotel accommodations (RR 1.30 [1.10–1.53], *P* < 0.01), travel to tropical South America (RR 1.34 [1.09–1.64], *P* < 0.01), and duration of travel (RR 1.00 [1.00–1.01], *P* < 0.01). The use of doxycycline for malaria prophylaxis is associated with lower TD risk, suggesting increasing bacterial enteropathogen susceptibility similar to previous observations. Doxycycline selection for antimalarial chemoprophylaxis may provide additional traveler benefit in infection prevention.

## INTRODUCTION

Travelers’ diarrhea (TD) is the most common illness experienced by international travelers and can occur during travel to any part of the world.^1,2^ Travel destination heavily influences the incidence rate of TD. Travelers to developing countries have an incidence rate of 20% per 2-week stay, whereas those traveling to low-risk areas (United States, Western Europe, and Australia) have a < 8% per 2-week stay TD incidence.^3^ Although mortality from TD is low, TD can cause significant incapacitation both during and after travel.^3^ Thirty percent of travelers to high-risk areas can become incapacitated from TD and may not be able to participate in planned activities for up to 24 hours.^3^ In addition, the evidence increasingly suggests a link between TD and the development of irritable bowel syndrome (IBS).^4^

With the high incidence of TD among international travelers and its associated morbidity, it is important to counsel travelers on TD prevention measures. Currently, TD prevention strategies are focused on food and water restrictions and chemoprophylaxis.^5,6^ Currently, there is no antibiotic with an approved indication for TD prophylaxis. In the previous two decades, rifaximin, a nonabsorbable antibiotic with a favorable safety profile, has demonstrated moderate efficacy in TD prevention.^5^ In the 1970s–1980s, doxycycline was commonly prescribed as TD prophylaxis. Multiple studies showed that taking daily doxycycline during travel significantly reduced the incidence of TD.^7,8^ By the mid-1980s, doxycycline resistance began to emerge, especially in enterotoxigenic *Escherichia coli* (ETEC).^9–11^ Therefore, the 1985 National Institute of Health Consensus Statement on TD recommended early treatment strategies for TD over the use of prophylactic antibiotics. Furthermore, they did not recommend doxycycline as an early treatment strategy.^12^

Doxycycline is still commonly used for malaria prophylaxis among international travelers. With changes in resistance patterns of enteric pathogens, it is unclear what effect doxycycline currently has on the development of TD.^9–11^ We examined a cohort of travelers who received various malaria-preventive strategies, seeking to evaluate whether the use of doxycycline as malaria prophylaxis was associated with a decreased risk of TD during international travel.

## MATERIALS AND METHODS

### Study design.

This study was a subset of the larger TravMil study: Deployment and Travel-Related Infectious Disease Risk Assessment, Outcomes, and Prevention Strategies among Department of Defense (DoD) Beneficiaries, approved by the Uniformed Services University of the Health Sciences Institutional Review Board (Bethesda, MD). TravMil is a prospective observational cohort of DoD beneficiaries traveling outside the continental United States for ≤ 6.5 months. Consenting adults were enrolled pretravel at six military travel centers (Walter Reed National Military Medical Center, Bethesda, MD; Brooke Army Medical Center, San Antonio, TX; Naval Medical Center, Portsmouth, VA; Madigan Army Medical Center, Tacoma, WA; Naval Medical Center, San Diego, CA; and Landstuhl Regional Medical Center, Landstuhl, Germany) and in the pre-deployment setting between January 2010 and August 2018. Eligible travelers were limited to DoD beneficiaries (e.g., active duty military personnel, military retirees, United States Public Health Service, Department of State employees, and military-dependent family members). Individuals were required to be traveling outside of the continental United States, Puerto Rico, Guam, Samoa, the U.S. Virgin Islands, western or northern Europe, Canada, or New Zealand for at least part of their itinerary. Military deployers were prescribed malaria prophylaxis in accordance with the military guidelines for their region of travel. Current DoD guidance recommends atovaquone–proguanil as the malaria prophylaxis for Africa. For all other regions, either doxycycline or atovaquone–proguanil can be prescribed at the discretion of their provider. Non-deployer travelers were prescribed malaria prophylaxis at the discretion of their provider.

### Survey data.

Pretravel enrollees completed a pretravel survey recording their demographic and travel characteristics. Prescriptions for malaria prophylaxis were abstracted from the clinic encounter. Travelers completed an illness diary during travel, recording the number of loose stools, abdominal pain, fever, nausea, and/or vomiting. Travelers’ diarrhea was defined as three or more loose stools in a 24-hour period or two loose stools in a 24-hour period, and one or more of the following: nausea, vomiting, abdominal pain, fever, or bloody stool. Episodes of TD were analyzed for each registered trip. For each registered trip which could include multiple geographic regions, the traveler was prescribed doxycycline, other, or no malaria prophylaxis for the duration of that trip.

### Statistical analysis.

Univariate and multivariate analyses were performed to determine which trip and traveler characteristics, including use of malaria prophylaxis, were associated with the development of TD. For univariate and multivariate analyses, a Poisson regression with robust error variance was used to estimate the RR of our count data. A robust error variance allowed for a tighter CI. Selected variables with a *P*-value of < 0.05 on univariate analysis underwent a Poisson regression for multivariate analysis to assess independent risk factors. This allowed for exclusion of highly correlated covariate and mutually exclusive variables. Results of multivariate analysis were reported with the RR with 95% CIs. Statistical significance was defined as a *P*-value of < 0.05. Statistical analysis was performed with SPSS software (IBM SPSS Statistics version 22, Chicago, IL).

## RESULTS

### Baseline characteristics.

A total of 3,227 trips with 2,803 travelers were analyzed (Table 1). The majority of travelers were male (62.1%), with a median age of 39 years (interquartile range [IQR] 27,59) and a median travel duration of 19 days (IQR 12,49). Caucasian (70.7%) was the most commonly reported race. Twenty-five percent of travelers were aged ≥ 60 years, and 27% who developed TD were aged ≥ 60 years. For malaria prophylaxis, 20% were prescribed doxycycline, 50% prescribed another form of malaria prophylaxis (89% of these were atovaquone–proguanil), and 30% were not prescribed malaria prophylaxis. Travelers’ itineraries could include more than one region of travel. Forty-five percent traveled to Southeast (SE) Asia/Pacific Islands, 32% traveled to Africa, and 2% traveled to tropical South America. The cumulative incidence of TD was 17%, with an incidence rate of 53 per 1,000 travel days. Of the 406 trips where travelers developed TD and were prescribed malaria prophylaxis, the most common countries where TD occurred were Peru (*n* = 58, 14.3%), Vietnam (*n* = 38, 9.3%), India (*n* = 29, 7.1%), and Honduras (*n* = 28, 6.9%).

**Table 1:** Traveler demographics, malaria prophylaxis prescribed, destinations, and incidence of TD

Characteristic	All (*N* = 3,227)	Non-deployment travel (*N* = 2,154)	Deployment (*N* = 1,073)
Gender, male, *n* (%)	2,004 (62)	1,106 (52)	898 (84)
Median age (years)	39 (IQR 27,59)	51 (IQR 33,65)	28 (IQR 23,35)
Median duration of travel (days)	19 (IQR 12,49)	16 (IQR 11,27)	34 (IQR 16,84)
Race, *n* (%)			
African American	345 (11)	244 (11)	101 (9)
Caucasian	2,252 (71)	1,508 (70)	744 (69)
Military status, *n* (%)			
Active duty	1,658 (51)	585 (27)	1,073 (100)
Dependent	1,454 (45)	1,454 (68)	–
Malaria prophylaxis prescribed, *n* (%)			
Doxycycline	644 (20)	174 (8)	470 (44)
Other*	1,623 (50)	1,235 (57)	388 (36)
None	960 (30)	745 (35)	215 (20)
Destinations, *n* (%)			
Africa	1,033 (32)	661 (31)	372 (35)
Southeast Asia/Pacific Islands	1,255 (39)	774 (36)	481 (45)
Caribbean, Mexico, and Central America	704 (22)	583 (27)	121 (11)
Tropical South America	314 (10)	291 (14)	23 (2)
Multiple regions	1,013 (31)	519 (24)	494 (46)
TD cumulative incidence, *n* (%)	562 (17)	502 (23)	60 (6)

IQR = interquartile range; TD = travelers’ diarrhea.

* 89% atovaquone–proguanil.

More than one purpose of travel and multiple types of travel accommodations could be reported (Table 2). The most common purposes of travel were military (46%) and vacation (40%). Only 3% of travelers who traveled for military purposes were in non-active duty (representing government employees/contractors). The most common travel accommodation was hotel (71%).

**Table 2:** Purpose of travel, travel accommodations, and type of travel location*

Characteristic	All (*N* = 3,227), *n* (%)	Non-deployment travel (*N* = 2,154), *n* (%)	Deployment (*N* = 1,073), *n* (%)
Purpose of travel			
Military	1,468 (46)	395 (18)	1,073 (100)
Vacation	1,282 (40)	1,282 (40)	–
Missionary	427 (13)	427 (13)	–
Medical	148 (5)	148 (5)	–
Travel accommodations			
Hotel	2,295 (71)	1,814 (84)	481 (45)
Dormitory	879 (27)	181 (8)	698 (65)
Military	198 (6)	198 (6)	–
Private	398 (12)	398 (12)	–
Type of location			
Rural	1,752 (54)	1,036 (48)	716 (67)
Safari	251 (8)	251 (8)	–

* Adoption, business, adventure, teaching, and camping purpose of travel were not included because of the limited number of travelers in these categories.

Data on dietary habits were available for 70% of trips with military accommodations. On these trips, 8.6% of travelers dined in only military facilities, 24.5% dined in military and outside facilities, and 66.9% dined in only outside facilities. There were 42 cases of TD within this group. There was one case of TD (2.4%) in a traveler who ate only at military facilities versus 23.8% who ate at both military and outside facilities and 73.8% who ate at only outside facilities (*P* = 0.2).

Of those trips that required malaria prophylaxis (*n* = 2,267), self-reported compliance data were available for 84% of trips during the study period. Compliance was defined as completing all malaria prophylaxis during and after travel as prescribed. For travelers who were prescribed doxycycline (*n* = 552), 70% reported compliance compared with 90% in the other malaria prophylaxis group (*P* < 0.01). Data on reasons for noncompliance were not collected.

### Travelers’ diarrhea association analysis.

On univariate analysis of travel characteristics (Table 3), female travelers, travelers to tropical South America, and increased duration of travel were associated with increased risk of developing TD. Vacation, missionary travel, hotel accommodations, and medical support missions were also associated with increased risk of developing TD. On univariate analysis, use of doxycycline for malaria prophylaxis (compared with combined group of use of other or no prophylaxis), travel to multiple regions, military purpose of travel, and dormitory accommodations were associated with decreased risk of TD. On univariate analysis, the RR of TD taking doxycycline for malaria prophylaxis versus another agent for malaria prophylaxis was 0.45 (0.35–0.58), *P* < 0.01. The univariate RR of TD taking doxycycline versus no malaria prophylaxis was 0.59 (0.34–0.74), *P* < 0.01. In addition to doxycycline, gender, military purpose of travel, travel to tropical South America, hotel accommodation, and duration of travel were chosen as variables for multivariate analysis. Gender and hotel accommodation were selected because they had a strong effect on the univariate analysis, and hotel accommodations represented a large proportion of the study population. Military purpose of travel was chosen as this is one of the key populations in the TravMil group and to ensure that doxycycline use and military travel were both considered in the model simultaneously. Travel to tropical South America was chosen for multivariate analysis as it was a distinct geographic region that was significantly associated with TD on univariate analysis. On multivariate analysis (Table 4), use of doxycycline (0.62 [0.47–0.82], *P* < 0.01) and military purpose of travel (0.57 [0.47–0.7], *P* < 0.01) remained significantly associated with decreased risk. On multivariate analysis (1.28 [1.09–1.5], *P* < 0.01), the median duration of travel (days) (1.0 [1.0–1.01], *P* < 0.01), travel to tropical South America (1.34 [1.09–1.64], *P* < 0.01), and hotel accommodations (1.3 [1.1–1.53], *P* < 0.01) were associated with increased risk.

**Table 3:** Univariate analysis of characteristics and RR of TD

Characteristic	TD	No TD	Univariate RR (95% CI)	*P*-value
Malaria prophylaxis, *n* (%)				
Doxycycline	62 (10)	582 (90)	0.5 (0.38–0.64)	< 0.01
Other or none	500 (19)	2,083 (81)	Ref	–
Gender, *n* (%)				
Male	281 (14)	1,723 (86)	Ref	–
Female	269 (22)	954 (78)	1.56 (1.34–1.81)	< 0.01
Age (years)	44 (30,62)	38 (26,58)	–	< 0.01
Median duration of travel (days)	19 (13,34)	9 (12,43)	–	0.61
Destinations, *n* (%)				
Africa	180 (17)	853 (83)	1.00 (0.85–1.18)	0.99
Southeast Asia/Pacific Island	207 (17)	1,048 (83)	0.92 (0.78–1.07)	0.27
Tropical South America	81 (26)	233 (74)	1.48 (1.21–1.82)	< 0.01
Mexico/Caribbean/Central America	132 (19)	572 (81)	1.06 (0.88–1.28)	0.49
Multiple regions	131 (13)	882 (87)	0.66 (0.55–0.80)	< 0.01
Purpose of travel, *n* (%)				
Military	168 (11)	1,300 (89)	0.51 (0.43–0.60)	< 0.10
Vacation	279 (22)	1,003 (78)	1.50 (1.28–1.74)	< 0.01
Missionary	117 (27)	310 (73)	1.72 (1.44–2.06)	< 0.01
Medical	47 (32)	101 (68)	1.9 (1.48–2.43)	< 0.01
Travel accommodations, *n* (%)				
Hotel	469 (20)	1,826 (80)	1.43 (1.22–1.68)	< 0.01
Dormitory	88 (10)	791 (90)	0.50 (0.40–0.61)	< 0.01
Military	54 (27)	144 (73)	1.62 (1.28–2.06)	< 0.01
Private	128 (22)	458 (78)	1.32 (1.12–1.58)	< 0.01
Type of location, *n* (%)				
Rural	291 (17)	1,461 (83)	0.92 (0.78–1.05)	0.18
Safari	60 (24)	191 (76)	1.42 (1.12–1.79)	< 0.01

TD = travelers’ diarrhea.

**Table 4:** Multivariate analysis of characteristics and their RR of travelers’ diarrhea

Characteristic	Multivariate RR (95% CI)	Multivariate *P*-value
Doxycycline use	0.62 (0.47–0.82)	< 0.01
Female gender	1.28 (1.09–1.5)	< 0.01
Median duration of travel (days)	1.0 (1.0–1.01)	< 0.01
Tropical South America	1.34 (1.09–1.64)	< 0.01
Military travel	0.57 (0.47–0.7)	< 0.01
Hotel accommodations	1.30 (1.1–1.53)	< 0.01

A further analysis of the use of doxycycline by region and associations with TD was performed. In our study, the region with the highest overall TD rates was Latin America at 20%. When this group was further subdivided into tropical South America, and the overall TD rate was 25%. There were similar rates of TD in Africa and Asia at 17% and 16%, respectively. When Asia was further subdivided into SE Asia, the TD rate was 13%. There was a decreased RR of TD when taking doxycycline and traveling to Latin America (0.85 [0.8–0.91], *P* = 0.01) and SE Asia (0.89 [0.85–0.94], *P* < 0.01). However, there was no statistically significant decreased RR of TD in the doxycycline groups in travelers to Africa and tropical South America, possibly secondary to the limited number of travelers on doxycycline in these groups (*N* = 75 and *N* = 13).

### Military subgroup analysis.

Because 82% of the doxycycline group were traveling for a military purpose, a subgroup analysis was performed (Table 5). A multivariate Poisson performed with the same variables as earlier, with the exception of travel to tropical South America because of the limited number of military travelers visiting this destination showed that doxycycline continued to have a statistically significant decreased RR of TD (0.46 [0.32–0.66], *P* < 0.01). Female gender (1.69 [1.25–2.3], *P* < 0.01), hotel accommodations (1.62 [1.25–2.3], *P* < 0.01), and longer duration of travel (1.01 [1.00–1.01], *P* < 0.01) continued to have a statistically significant increased RR of TD.

**Table 5:** Multivariate analysis of characteristics and their RR of travelers’ diarrhea in military subgroup

Characteristic (*N* = 1,468)	Multivariate RR (95% CI)	Multivariate *P*-value
Doxycycline use	0.46 (0.32–0.66)	< 0.01
Female gender	1.69 (1.25–2.29)	< 0.01
Median duration of travel (days)	1.01 (1.0–1.01)	< 0.01
Hotel accommodations	1.62 (1.19–2.22)	< 0.01

## DISCUSSION

Our evaluation of the use of doxycycline as malaria prophylaxis and risk of TD among international travelers demonstrates a negative association between doxycycline used as malaria prophylaxis and incidence of TD in international travelers. Given the widespread prevalence of TD in international travelers, as well as the limitations and unclear efficacy of dietary restrictions for prevention, and limited options for chemoprophylaxis, these findings warrant consideration. Rifaximin is approved for the prevention of TD in selected populations.^5^ Meta-analysis shows that rifaximin has a decreased RR of developing TD (RR 0.41 [0.3–0.56], *P* < 0.01)^13^ compared with our study showing doxycycline RR of 0.62 (0.47–0.82), *P* < 0.01. Although these observational data do not derive from a randomized controlled trial, the findings support doxycycline effectiveness in TD prevention.

Identification of the precise etiologic agent of TD has historically been difficult. With advances in etiologic determination using molecular-based diagnostics, there is increased capability to identify the causative agent of TD.^14,15^ Even with these advances in technology, studies still show that 25–30% of samples will have no pathogen identified.^3,16^ When a pathogen is identified, diarrheagenic *E. coli* (DEC), primarily ETEC and EAEC, are the most common etiologies globally with some regional variation where other agents are identified, such as in Southeast Asia where *Campylobacter* is common.^16^ Other TD bacterial pathogens of concern such as *Shigella*, *Salmonella*, and non-cholera *Vibrio* species only represent < 10% of TD globally.^3,16^

Doxycycline use as a prophylactic antibiotic against TD pathogens, especially DEC, has been well documented historically. In 1976 and 1977, randomized double-blinded placebo-controlled trials in Peace Corps volunteers in Kenya and Morocco showed that a 100-mg daily dose (malaria prophylaxis dose) of doxycycline offered 83–86% protection against TD.^7,8^ In 1980, another randomized double-blinded placebo-controlled trial in Peace Corps volunteers in Thailand using the 100-mg daily dose of doxycycline did not show statistically significant protection from TD.^10^ Similar studies in 1980 and 1981 in Honduras and Mexico did show a statistically significant protective effect of doxycycline against TD, respectively.^11,17^ Notably, in those studies that showed a protective effect of doxycycline, most stools of symptomatic participants grew ETEC.^7,8,11,17^ The Honduras study which showed the lowest level of statistically significant protection with doxycycline (68% versus 80% in all other studies) also showed 54% of the ETEC isolates in the doxycycline group were resistant to doxycycline. Interestingly, the subjects that developed TD while on doxycycline and grew doxycycline-resistant ETEC had less severe symptoms than subjects with TD in the placebo group.^11^ These studies show the historic microbiologic basis for doxycycline having a protective effect against TD.

To further explore the decreased risk of TD seen with doxycycline in our study, it is important to review the evolution of doxycycline resistance. In 1983, the MIC_90_ of doxycycline against *Salmonella* was 64 µg/mL, *Shigella* 16 µg/mL, *Campylobacter* 8 µg/mL, and ETEC 32 µg/mL.^18^ Given these MIC_90s_, doxycycline’s long half-life, and high intraluminal concentration, doxycycline was still considered to be an efficacious prophylactic medication for TD. By 1997, there was an increase in the MIC_90s_ of all of these enteric pathogens against doxycycline. The MIC_90_ of doxycycline against *Salmonella* and *Shigella* was now 128, ETEC 64, and *Campylobacter* 64. In addition, this 1997 study showed regional differences in antibiotic susceptibility.^19^ This study showed the increasing levels of doxycycline resistance and that prophylactic use of doxycycline may only be efficacious against certain pathogens in distinct parts of the world. A more recent study looked at the resistance patterns of ETEC in Bangladesh over time. From 2005 to 2009, the prevalence of doxycycline resistance among ETEC isolates increased from 33% to 44%.^15^

Among common causes of TD, ETEC remains the pathogen with the highest likelihood of susceptibility to doxycycline. Notably, there is not an even geographic distribution of ETEC. According to the Global Burden of Disease Study 1990–2016, the global burden of ETEC is approximately 30.1 cases per 1,000 person years. Of note, one of the highest regions of ETEC incidence was Andean Latin America (Bolivia, Peru, and Ecuador) at 81.1 cases per 1,000. The Global Burden of Disease Study also shows that for high TD incidence regions, Southeast Asia has lower incidence of ETEC (30.8/1,000) than other high-burden regions such as sub-Saharan Africa (52.8/1,000).^20^ In our study, a known region with a high ETEC burden, Latin America, was shown to have a statistically significant decreased RR of TD for travelers on doxycycline (0.85 [0.8–0.9.91], *P* = 0.01). The region of tropical South America which includes Andean Latin America did not show a statistically significant decreased RR of TD on doxycycline (0.87 [0.69–1.110], *P* = 0.53), which may be secondary to the limited amount of travelers on doxycycline. A recent analysis from the same TravMil cohort used TaqMan Array Card testing of self-collected stool smears on Whatman FTA Elute Cards to determine the pathogen-specific epidemiology of TD and found ETEC to be the most commonly associated pathogen and in similar proportions across geographic areas of the world.^21^ The reasons for differing the RR of TD in travelers using doxycycline in our study could include differing rates of ETEC, differing rates of doxycycline resistance, or limited statistical power for certain regions.

Nonmicrobial effects of doxycycline may also contribute to a decreased risk of TD. Diarrhea is a highly inflammatory process. Murine models have shown that T-cell activation of tumor necrosis factor (TNF), interleukin (IL)-1, IL-6, and other cytokines induces fluid accumulation in the small bowel, leading to diarrhea.^22^ In addition, studies in children have shown high serum levels of TNF-α, IL-6, and IL-10 after having viral and/or bacterial gastroenteritis.^23,24^ Tetracyclines have a regulatory influence on the immune system and inflammatory pathways.^25,26^ Doxycycline has been shown to inhibit activation of macrophages which in turn inhibits TNF-α, IL-1b, IL-6, and other pro-inflammatory proteins. Tetracyclines also inhibit lipopolysaccharide-induced nitric oxide production. Nitric oxide production has been implicated in the pathophysiology of sepsis.^25^ Doxycycline also has antioxidant properties, thereby reducing tissue destruction.^27^ Therefore, the decreased RR of TD seen in our study could be theoretically related to doxycycline, reducing the inflammatory cascade associated with diarrhea independent of direct antimicrobial effects.

Several additional factors described in this study as being associated with risks of TD are consistent with previously published data. Latin America has been well established as a high-risk TD incidence region, with attack rates approximating 39.4 cases per 100 person-months.^28–30^ Associations with gender and development of TD have been previously reported.^3,31^ GeoSentinel data have shown that females have a higher rate of developing acute diarrhea, chronic diarrhea, and IBS.^31^ Although other studies have not shown difference in rates of TD, they found that females are more likely to seek medical care for their TD.^3^

Interestingly, hotel stay has been previously reported as being associated with increased risk of TD.^3,32^ Some associations between hotel stay and TD have been secondary to an outbreak.^32^ However, Steffen et al.^3^ showed that hotel stay in high-risk geographic regions for TD was associated with increased risk of TD. In addition, this study showed that all-inclusive resorts had an overall higher rate of TD than when participants had to pay for meals at their hotel.^32^ Although our study did show an increased RR of TD in subjects staying at a hotel, it is unknown how many participants stay in all-inclusive resorts in our study.

Our study had multiple limitations. First, our study collected observational data with self-reported enteric symptoms as well as adherence to malaria prophylaxis. The lack of TD pathogen identification limits a direct assessment of doxycycline breakthroughs. Our study did not record confounding medical conditions or concomitant medications that could predispose travelers TD or influence providers’ decision on which malaria prophylaxis to prescribe. Dietary habits and the risk for TD were not explored in non-deployment travel portion of this study as previous TravMil studies have not shown an association between TD and dietary habits.^33^ Finally, although we attempted to control for geographic variability to some extent in the multivariate analysis, it may be that areas of travel in which prescribers favored doxycycline also may be lower-risk itineraries with lower rates of TD.

Previous studies have shown that there is seasonality in TD. Season affects both the frequency of TD seen and pathogens causing TD.^34^ In our study, the median travel duration was 19 days. However, on military deployment trips, the median duration was 34 days. In addition, subjects frequently traveled to multiple locations, 31%, and, therefore, portions of their travel could be in different regions of exposure. Because so many trips involved changes in season (including changes in seasonality based on geography), we did not explore seasons of travel and their effect on TD.

Because the aim of the study was to look at the risk factors associated with TD, the use of antibiotics for TD self-treatment was not addressed in this study. In addition, this study was designed to show the effect of doxycycline as malaria prophylaxis on TD during an acute period of time. One of the limitations of this study is that long-term effects of the use of doxycycline were not evaluated. TravMil is currently evaluating risk factors for extended B-lactamase (ESBL) *E. coli* colonization after military travel by looking at region of travel, malaria prophylaxis, and antibiotics used for TD self-treatment. Initial data from this study did not show the use of doxycycline as malaria prophylaxis or the use of antibiotics for self-treatment of TD to be associated with ESBL colonization.^35^ Studies in civilian populations have shown association between antibiotic use for self-treatment of TD and ESBL colonization.^36,37^ However, when one study looked at antibiotics used for reasons other than self-treatment of TD and antimalarial chemoprophylaxis, there was no association with ESBL colonization.^36^

Doxycycline for malaria prophylaxis requires an additional 28 days of medication after returning from travel. These long courses of antibiotics also have the potential to alter the microbiome and cause antibiotic induce *Clostridioides difficile*. There are mixed data on the correlation between doxycycline and *C. difficile*. This case-controlled study of hospitalized patients studied the relationship between certain antibiotics and hospital-acquired *C. difficile* infection (CDI). Doxycycline had a slightly reduced risk of CDI with an odds ratio of 0.41.^38^ However, there have been case series of travelers taking doxycycline for malaria prophylaxis developing CDI.^39^ With doxycycline’s ability to highly concentrate in the bowel, there is concern for this antibiotic altering the gastrointestinal microbiome.^40^ Studies of subjects taking long courses of doxycycline (more than 1 year in these studies) have shown decreased diversity in the gastrointestinal microbiome.^41,42^

One of the greatest strengths of our study is the large sample size, with 3,227 trips recorded including various approaches to malaria prophylaxis, which allowed for comparison of doxycycline TD prophylaxis effectiveness. Furthermore, our military subgroup analysis showed that the use of doxycycline was still associated with a statistically significant decrease in TD risk, further strengthening our findings and decreasing the likelihood that the decreased risk may be attributed to some other military-specific aspect of travel.

## CONCLUSION

The cumulative incidence of TD in international travelers who were taking doxycycline for malaria prophylaxis was decreased compared with those taking other or no prophylaxis. This effect may be because of increasing bacterial enteropathogen susceptibility to doxycycline against diarrheagenic *E. coli*, similar to observations from over 30 years ago, and potential anti-inflammatory effects. Further prospective studies should be performed to establish if there are any additional benefits of using doxycycline as malaria prophylaxis.
